# Microbial phylogenetic profiling with the Pacific Biosciences sequencing platform

**DOI:** 10.1186/2049-2618-1-10

**Published:** 2013-03-04

**Authors:** Erin B Fichot, R Sean Norman

**Affiliations:** 1 Department of Environmental Health Sciences, University of South Carolina, Columbia, SC, USA

**Keywords:** PacBio, Pacific Biosciences, Amplicon, 16S rRNA, Circular consensus sequence (ccs), Microbial diversity

## Abstract

High-throughput sequencing of 16S rRNA gene amplicons has revolutionized the capacity and depth of microbial community profiling. Several sequencing platforms are available, but most phylogenetic studies are performed on the 454-pyrosequencing platform because its longer reads can give finer phylogenetic resolution. The Pacific Biosciences (PacBio) sequencing platform is significantly less expensive per run, does not rely on amplification for library generation, and generates reads that are, on average, four times longer than those from 454 (C2 chemistry), but the resulting high error rates appear to preclude its use in phylogenetic profiling. Recently, however, the PacBio platform was used to characterize four electrosynthetic microbiomes to the genus-level for less than USD 1,000 through the use of PacBio’s circular consensus sequence technology. Here, we describe in greater detail: 1) the output from successful 16S rRNA gene amplicon profiling with PacBio, 2) how the analysis was contingent upon several alterations to standard bioinformatic quality control workflows, and 3) the advantages and disadvantages of using the PacBio platform for community profiling.

## Background

**Figure 1:**
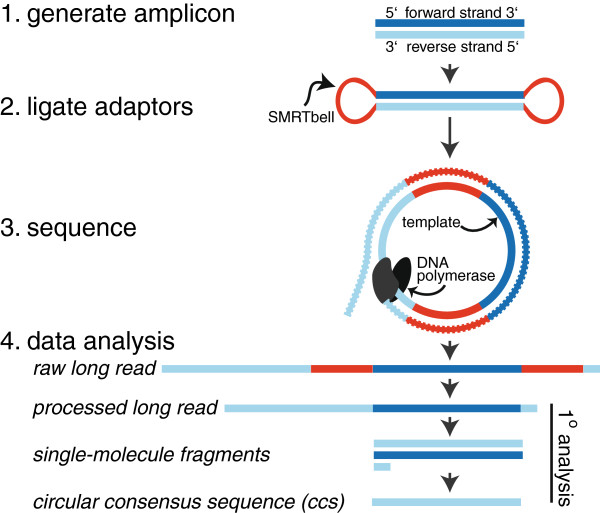
**Illustration of PacBio sequence generation.** Adaptors (SMRTbells) are first ligated to each amplicon, and after a sequencing primer is annealed to the SMRTbell template, DNA polymerase is bound to the complex. This polymerase-amplicon-adaptor complex is then loaded into zero-mode waveguides (ZMWs) where replication occurs, producing nucleotide-specific fluorescence. Circular consensus sequencing (ccs) allows the polymerase to repeatedly replicate the circularized strand, producing one long read with randomly distributed errors [9]. Post-run, the SMRTbell sequences are bioinformatically trimmed away, single-molecule fragments are aligned, and a consensus sequence is generated. The single-molecule coverage and accuracy of resulting ccs reads are amplicon- and read-length dependent, with smaller amplicons and longer reads giving higher single-molecule coverage and thus higher ccs read accuracy.

The phylogenetic profiling of microbial communities using 16S rRNA gene amplicon sequencing is a routine practice in microbial ecology. High coverage of diversity and highly accurate sequence reads are required for these studies, while long reads can enhance phylogenetic resolution. Roche’s 454 Genome Sequencer has been the dominant sequencing platform for 16S rRNA gene amplicon surveys because of its longer read length (700 to 800 bp versus Illumina’s 2 × 100 bp), but Illumina is also used because of the large number of reads generated (≤1.5 billion reads per run) [1,2]. Pacific Biosciences (PacBio) is a less expensive platform (per run) and produces much longer reads (3,000 to 15,000 bp [1]) without a library preparation amplification step, but a recent review found that PacBio was in theory the least suitable out of the major high-throughput sequencing platforms available for phylogenetic profiling [1], mainly due to its low accuracy [3]. Since phylogenetic profiling requires high read accuracy, low quality reads are problematic, but this issue can be alleviated through the use of PacBio circular consensus sequencing (ccs). For this, ligated hairpin structures allow sequencing-by-synthesis to occur on circularized amplicons, such that long reads provide high single-molecule coverage and thus improved accuracy (Figure 1).

**Figure 2:**
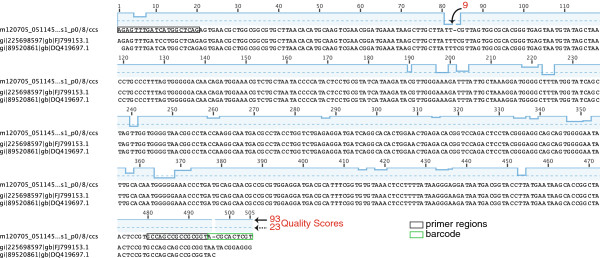
**Clustlw alignment of a typical PacBio ccs read from Marshall *et al*. [**[4]**] with the top two blastn matches (nr).** The quality score is shown graphically (log scale) above the PacBio ccs read (m12705_051145…s1/8/ccs) and ranges from 9 (positions 81 to 84) to 93, with an average of 78.6. The dashed line indicates a quality score of 23, equivalent to an accuracy of 99.5%. Note that regions with low quality scores are associated with homopolymers, but homopolymers do not always have low quality scores. The processed long read from which this specific ccs sequence was generated was 4944 nt long, with a quality score that ranged from 0 to 15 and averaged 10.3. The length equates to approximately 9.5× single-molecule coverage.

A recent study using PacBio to characterize an electrosynthetic microbiome demonstrated that this platform provided sequences well suited for genus-level discrimination (through taxonomic binning) of a mixed microbial community [4]. The size of the 16S rRNA gene V1-V3 (approximately 515 bp, bacteria) or V2-V3 (approximately 400 bp, archaea) amplicons gave sufficient single-molecule coverage (using C2 chemistry and a 45 min movie length) to produce full-amplicon-length ccs reads with an average Phred quality score of 60 (1 in 1,000,000 probability of an incorrect call at each base) after quality control (QC) (Figure 2). Running a multiplex of four samples (bacteria and archaea amplicons for each of two samples) in two PacBio cells yielded approximately 70,000 full-amplicon-length sequence reads (after approximately 35% sequence removal for QC) for less than USD 1,000, with >91% archaeal or bacterial coverage at the genus level (0.05 operational taxonomic unit (OTU)-level) for each sample.

Bioinformatic workflows used to preprocess raw sequences before phylogenetic analysis have quality control features designed to reduce sequencing or PCR errors in the dataset. For example, workflows remove reads that contain: an ambiguous base call, an average quality score below a threshold, multiple mismatches to a primer/barcode sequence, fewer than a specified number of bases, or chimeras [5]. As this workflow was not wholly sufficient for use with PacBio-generated data output [4], the necessary alterations are discussed further in the text, all of which can be executed with open-source software such as mothur [6] or QIIME [7].

## Main text

### Strand orientation

Unlike sequences originating from 454, the user cannot control which template strand (+/−) is sequenced in PacBio. Therefore, strand orientation must be recognized and unified if alignments or OTU-based analyses are to be performed.

### Retrieving quality scores

The overall quality score (Phred + 33) can be retrieved from the fastq output with the aid of scripts that translate the ASCII code into a Phred score. In addition to scripts available in mothur and QIIME, freely available Perl scripts, such as fq_all2std.pl, can be used with the std2qual command to retrieve Phred scores and sequences from PacBio fastq output files.

### Ambiguous bases

In 454 sequences, a base call receiving a quality score of 0 is assigned the ambiguous base designation ‘N’. However, in PacBio sequences, a base call receiving a quality score of 0 is assigned an actual nucleotide. Therefore, culling reads based on ambiguous base calls does not remove the intended reads. Instead, scripts need to search the quality score file in order to remove these reads.

### Stretches of low quality

Quality decreases toward the end of 454-generated reads [8], but because of the nature of generating a ccs read from a long read with randomly distributed errors [9], PacBio ccs reads are not only the full amplicon length, but the read quality does not positionally decrease (Figure 2). Instead, regions of lower quality in PacBio sequences appeared to be homopolymer associated, but not all homopolymers had low quality (Figure 2). Removing sequences based on the read’s average quality score was not the most rigorous or appropriate way to remove ‘bad’ ccs sequences because the abundant, heretofore unseen high quality scores (≤93) of PacBio reads mask regions of lower quality. Using a rolling window approach to reduce errors (removing reads when the average quality score over a window of specified bases drops below a threshold) was used in Marshall *et al*. [4] with a window spanning twice the size of the average homopolymer. As seen in Figure 2, large window sizes include regions of extraordinarily high quality scores, thus masking low quality regions. Using small windows would remove sequences with legitimate homopolymers (Figure 2, see positions 364 to 388).

### Chimeras

**Figure 3:**
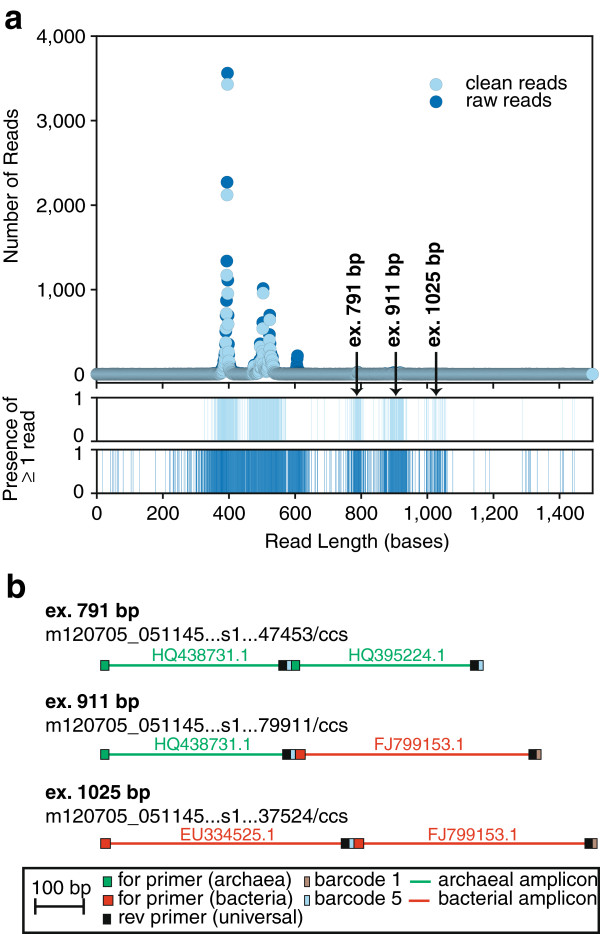
**Illustration of how initial preprocessing and chimera check did not remove ‘PacBio chimeras’.** (**a**) Read length distribution from one representative look of Marshall *et al.*[4] before and after initial sequence preprocessing, where a ‘look’ is the field-of-view used in fluorescence data collection. This particular look was from the sample containing multiplexed bacterial and archaeal amplicons from Day 91 of a microbial electrosynthesis cell: barcode 1 was from supernatant and barcode 5 from granules (read prefex m120705_051145…s1/…/ccs). Initial preprocessing removed reads with any of the following: average quality score <25, ambiguous base calls (‘N’), >8 homopolymers, length >350 bp, >1 mismatch to each primer, >1 mismatch to a barcode, or a chimera. After initial preprocessing, 1.5% of cleaned reads were ≥700 bp. (**b**) Motifs from representative reads (names listed with motif) of unexpected sizes are shown to scale, with the accession number to best blastn (nr) match to each segment written above segments.

**Figure 4:**
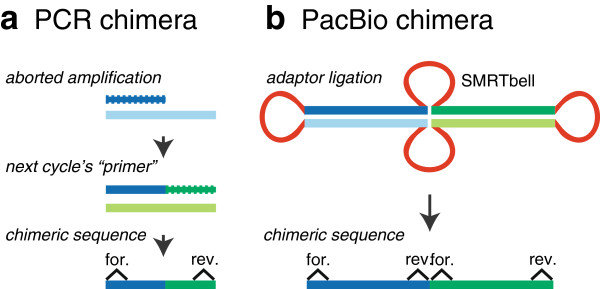
**Comparison of chimeras generated by PCR and those hypothetically generated during the PacBio library preparation.** (**a**) PCR-generated chimeras are typically created when an aborted amplicon acts as a primer for a heterologous template. Subsequent chimeras are about the same length as the non-chimeric amplicon and contain the forward (for.) and reverse (rev.) primer sequence at each end of the amplicon. (**b**) PacBio-generated chimeras originate during the adaptor ligation step. The length of these chimeras is measured in multiples of non-chimeric amplicon length, and they contain multiple forward and reverse primer sequences (found at the beginning and end of each segment).

Chimeras, sequences containing fragments from different templates, are well-known PCR artifacts [10]. Initial quality control workflows for the PacBio reads in Marshall *et al.*[4] contained a step to exclude chimeric sequences (UCHIME) [11]. However, sequences larger than expected made it through the pipeline (approximately 1% to 2% of preprocessed reads), and upon closer inspection, these reads were chimeras (Figure 3). Unlike PCR-generated chimeras, the heterologous fragments of these chimeras were full-length amplicons, complete with primer and barcode sequences, leading to the hypothesis that these chimeras were generated during the SMRTbell adaptor ligation step of PacBio library preparation (Figure 4). In light of this finding, sequences outside the expected amplicon size should be removed.

## Discussion

Depending on the diversity in the biological system being analyzed and the researcher’s resources/requirements (funds, phylogenetic resolution, coverage, number of samples, etc.), one sequencing platform may be more appropriate than another for phylogenetic profiling [1,12-15]. Illumina provides higher coverage than 454 or PacBio, but PacBio and 454 are advantageous when a higher phylogenetic resolution is needed (longer sequence reads). PacBio is also advantageous for labs with less resource because it could enable less expensive routes to data exploration without sacrificing phylogenetic resolution. In addition, PacBio’s relatively low cost per run may benefit studies that require only a few samples to be sequenced, where the cost per sample on other platforms can be prohibitive. Currently, several US academic institutions offer PacBio sequencing services and charge approximately USD 350 to USD 440 for library preparation and USD 200 to USD 400 for each cell used in sequencing. For the same price, neither Illumina’s GAIIx/HiSeq2000 nor 454 GS FLX is available, but several benchtop instruments provide sequencing services for run costs equivalent to PacBio [12].

In terms of systemic error, each platform also has advantages and disadvantages. Illumina and 454 have low error rates, but the errors are positional (they increase distally, with guanine-cytosine (GC) content, or with homopolymers) [8,9,15]. In contrast, PacBio has high error rates, but through the use of ccs reads and because errors are randomly distributed, the error rates are greatly reduced.

While no study has documented a head-to-head or *in silico* comparison of community amplicons sequenced with PacBio ccs and another platform such as Illumina or 454, there is evidence that PacBio may not add extensive platform-based bias to community profiles. In sequencing three microbial genomes containing either 19%, 50% or 69% GC content with PacBio, Carneiro *et al*. [9] found that the read coverage was relatively unaffected by GC content, with Quail *et al*. [15] finding similar results. Conversely, Ion Torrent, Illumina, and 454 all have a noticeable GC bias [15-17], but Aird *et al*. [18] attribute this to bias introduced in PCR amplification during the library preparation step. Unlike Illumina and 454, the library preparation for PacBio does not include an amplification step, which avoids this as a potential sequencer-based error source. On the other hand, one known bias of the PacBio platform is the preferential loading of shorter sequences into zero-mode waveguides (ZMWs, essentially ‘wells’), thus biasing the resulting community toward members having shorter sequences; but if amplicons are used, this bias is minimized. A comparison between platforms to determine PacBio-specific bias in community profiling is a necessary next step.

## Conclusion

Overall, the PacBio sequencing platform was sufficient for phylogenetic profiling of electrosynthetic microbiomes to the genus level with taxonomic binning [4]. The low read quality typical of PacBio was overcome by using circular consensus sequences (ccs). In addition, quality control workflows were adjusted for PacBio-specific issues, the most notable of which was the formation of ‘PacBio chimeras,’ features that are a potential artifact of PacBio library preparation but are not detected with UCHIME. Just as with every sequencing platform, future advances by PacBio in technology and chemistry will enable longer (hence more accurate and numerous) reads, while further understanding of PacBio biases will enable more accurate data for phylogenetic profiling.

## Abbreviations

ASCII: American standard code for information interchange;ccs: circular consensus sequences;GC: guanine-cytosine;OTU: operational taxonomic unit;PacBio: Pacific Biosciences;QC: quality control;rRNA: ribosomal RNA;SMRT: single-molecule real time;USD: United States dollar;ZMW: zero-mode waveguide

## Competing interests

The authors declare that they have no competing interests.

## Authors’ contributions

EF carried out the bioinformatic analyses and drafted the manuscript. RN participated in the design and coordination of the study and helped to draft the manuscript. All authors read and approved the final manuscript.

## Authors’ information

EF is research specialist and RN is a molecular microbial ecologist and assistant professor in the Environmental Health Sciences Department of the University of South Carolina.
